# Interaction of magnetite-based receptors in the beak with the visual system underlying 'fixed direction' responses in birds

**DOI:** 10.1186/1742-9994-7-24

**Published:** 2010-08-13

**Authors:** Roswitha Wiltschko, Dennis Gehring, Susanne Denzau, Onur Güntürkün, Wolfgang Wiltschko

**Affiliations:** 1 Fachbereich Biowissenschaften der J. W. Goethe-Universität, Siesmayerstraße 70, D-60054 Frankfurt am Main, Germany; 2 Abteilung Biopsychologie, Fakultät für Psychologie, Ruhr Universität Bochum, D-44780 Bochum, Germany

## Abstract

**Background:**

European robins, *Erithacus rubecula*, show two types of directional responses to the magnetic field: (1) compass orientation that is based on radical pair processes and lateralized in favor of the right eye and (2) so-called 'fixed direction' responses that originate in the magnetite-based receptors in the upper beak. Both responses are light-dependent. Lateralization of the 'fixed direction' responses would suggest an interaction between the two magnetoreception systems.

**Results:**

Robins were tested with either the right or the left eye covered or with both eyes uncovered for their orientation under different light conditions. With 502 nm turquoise light, the birds showed normal compass orientation, whereas they displayed an easterly 'fixed direction' response under a combination of 502 nm turquoise with 590 nm yellow light. Monocularly right-eyed birds with their left eye covered were oriented just as they were binocularly as controls: under turquoise in their northerly migratory direction, under turquoise-and-yellow towards east. The response of monocularly left-eyed birds differed: under turquoise light, they were disoriented, reflecting a lateralization of the magnetic compass system in favor of the right eye, whereas they continued to head eastward under turquoise-and-yellow light.

**Conclusion:**

'Fixed direction' responses are not lateralized. Hence the interactions between the magnetite-receptors in the beak and the visual system do not seem to involve the magnetoreception system based on radical pair processes, but rather other, non-lateralized components of the visual system.

## Background

In migratory birds, two types of directional responses to the magnetic field have been observed, namely normal *compass orientation *and, under certain unnatural light conditions, so-called *'fixed direction' responses *(for review, see [1]). Both these behaviors differ in many aspects; they originate in different magnetoreceptors based on different biophysical mechanisms: compass orientation, controlled by the avian inclination compass, is mediated by radical-pair processes [2-4] in the eye [5]; it is this system that provides birds with directional orientation to locate their home direction, migratory direction or any acquired directions. 'Fixed direction' responses, in contrast, are polar [1,6] and originate in the magnetite-based receptors in the upper beak [7-10]. Their behavioral significance is unclear, as they so far have been observed only under conditions that do not occur in nature. Normally the receptors in the beak appear to mediate magnetic 'map' information [11,12]; their additionally providing directing information might be a phylogenetic relict from an ancient compass mechanism that has now been replaced by the radical-pair mechanism in birds (see [1] for discussion).

Both responses-compass orientation as well as 'fixed directions'-are light-dependent, but also their light-dependency is fundamentally different. Compass orientation occurs under 'white' light (e.g. light composed more or less of the full spectrum) and under low intensity monochromatic light from the short-wavelength part of the spectrum up to 565 nm green [1]. If light conditions meet these requirements, the specific directions no longer depend on the light regime, but on the goal the birds are trying to reach. 'Fixed direction' responses, in contrast, have been observed under bright monochromatic light, bichromatic light with a long-wavelength component and in total darkness, and their manifestation depends on the ambient light regime [1]: different light conditions lead to headings in different directions: e.g. European robins, *Erithacus rubecula*, headed north under a combination of green-and-yellow light and south under blue-and-yellow light, regardless of season [1,13].

The observation that the 'fixed directions' depend on the ambient light regime was rather surprising, because one would not have expected magnetite-based receptors to be influenced by light. The findings mentioned above indicate connections with the visual system: obviously, the visual system is involved in controlling the specific 'fixed direction' emerging under a given light condition. This raises the question about the nature of this interaction. The magnetite-based receptors provide directing input, which, however, does not seem to tell birds compass directions as it cannot be used to locate the migratory direction. The ambient light conditions modify this input in a specific way, depending on the wavelengths or wavelengths-composition of the light, and make the birds head in a specific direction. Two types of interactions with the visual system seem possible: (1) the input from the receptors in the beak could interact with information from the magnetoreception system in the eye that normally provides birds with directional information, or (2) it could interact with other parts of the visual system. To answer this question, we made use of the finding that compass orientation, like many other features associated with the eyes and vision (for review, see [14]) is lateralized in favor of the right eye [5,15,16]. We tested birds monocularly, i.e. with one eye covered. If the 'fixed direction' responses were likewise found to be lateralized, this would suggest an interaction between the two magnetoreception systems, while finding the 'fixed direction' responses not lateralized would indicate interactions of the magnetite receptors with non-lateralized components of the visual system.

## Methods

The respective experiments were performed in Frankfurt am Main, Germany (50°08'N, 8°40'E), from 11 January to 21 February 2010, in the local geomagnetic field of 47 μT, 66° inclination.

### Test birds and test performance

The test birds were European robins, nocturnal migrants that breed in most parts of Europe and winter in the Mediterranean countries. They were caught as juvenile transmigrants, probably of Scandinavian origin, in September in the Botanical Garden in Frankfurt and kept over the winter. The photoperiod simulated the natural until beginning of December, when it was reduced to L:D 8:16 h. One week before the tests began, the light period was increased in two steps to L:D 13:11 to induce premature *Zugunruhe *(migratory restlessness) that allowed us to test the birds in spring migratory state already in January.

For the monocular tests, we used the same method as described by [5]: a small aluminum cap covered the eye, fixed to the bird's head with adhesive tape (Leukoplast). This cap was placed either over the right or the left eye immediately before the test and removed as soon as the test was over. Birds to be tested binocularly received no treatment.

All tests took place in wooden houses in the garden of the Zoological Institute where the geomagnetic field was largely undisturbed. Testing began when the light went off in the housing cages, and lasted about 1 h. The birds were tested one at a time in funnel cages lined with thermo-paper (Blumberg Systempapiere), where they left marks as they moved. Each funnel cage was placed in a light-proof cylinder isolating it from the others.

### Light conditions

For compass orientation, we used turquoise light; for a 'fixed direction' response, we choose the easterly one observed under a combination of turquoise-and-yellow light. Both these light conditions have been analyzed in detail in previous studies, and both have been found to show the typical characteristics of the respective type of response (see [1]).

The test lights were produced by light-emitting diodes (LEDs) mounted on a plastic disk that covered the top of the cylinder. Their light passed two diffusers before it reached the bird in the cage. Turquoise light had a peak wavelength of 502 nm (half band-width 486-518 nm) with an irradiance of 2.1 mW/m^2 ^corresponding to 5.3 10^15 ^quanta s^-1 ^m^-2 ^measured within the test cage; the turquoise-and-yellow light was a combination of the 502 nm turquoise light described above with 590 nm yellow light (half band-width 572-609 nm) and an irradiance of 1.8 mW/m^2 ^so that both components had equal quantal flux of 5.3 10^15 ^quanta s^-1 ^m^-2^. Each bird was tested three times in each condition (and in three others belonging to another study), alternating between the conditions in a pseudo-random sequence. The binocular test under 502 nm turquoise light served as control.

### Data analysis and statistics

For data analysis, the thermo-paper was removed, divided into 24 sectors, and the scratch marks in each sector were counted double blind. Recordings with a total of fewer than 35 scratches were excluded from the analysis and were repeated. From the distribution of the activity within the cage, the heading of the respective test was calculated, and from the three headings of each bird, we calculated the respective mean vector of that bird with the direction α_b _and the length r_b_. The mean headings α_b _of the 12 test birds were comprised in the grand mean vector for each condition, with the direction α_N _and the length r_N_, which were tested by the Rayleigh test for significant directional preferences [17]. The data sets under turquoise and turquoise-and-yellow light were compared by the Watson Williams Test for differences in direction [17]; the birds' orientation in the monocular conditions were compared with that in the binocular one under the same light regime with the non-parametric Mann Whitney U-test applied to the angular deviation of each bird's mean from the grand mean direction to test for differences in variance. From the vector length r_b _the median was calculated to reflect the intra-individual variance, and the data of the monocular tests were compared with those from the respective binocular sets using the Wilcoxon test for matched samples.

## Results

**Figure 1:**
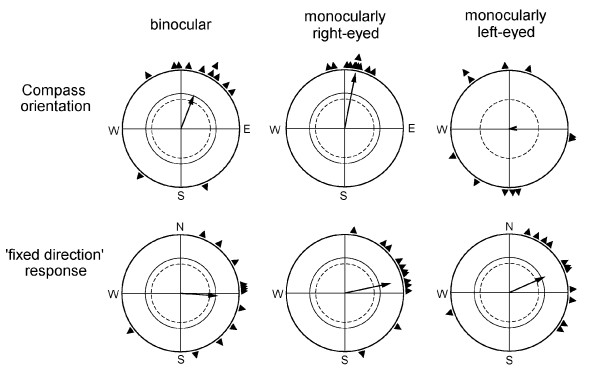
**Orientation of robins during spring migration**. Compass orientation was recorded under 502 nm turquoise light and the 'fixed direction' response under a combination of 502 nm turquoise and 590 nm yellow light. The triangles at the periphery of the circle indicate the mean heading of individual birds based on three recordings each, the arrows represent the grand mean vectors in relation to the radius of the circle = 1, with the two inner circles representing the 5% (dotted) and 1% significance border of the Rayleigh test [17].

The results are shown in Fig. 1, with Table 1 giving the numerical data and the statistical differences between conditions; Tables 2 and 3 list the behavior of the individual birds under turquoise and turquoise-and-yellow light, respectively. Under 502 nm turquoise light, the robins were oriented in their seasonally appropriate northerly migratory direction slightly east of north; under turquoise-and-yellow light, in contrast, they preferred easterly headings in a 'fixed direction' response that is significantly different from the migratory direction (F = 7.511, p < 0.05, Watson Williams test).

**Table 1:** Orientation of binocular and monocular robins: compass orientation and a 'fixed direction' response

Response	Light (nm)	Eyes	N	med r_b_	α_N_	r_N_	Δbi
Compass orientation	502	binocular	12	0.96	21°	0.62**	
	502	monocularly right-eyed	12	0.74 ^n.s.^	11°	0.97***	-10° *
	502	monocularly left-eyed	12	0.39 **	265°	0.04^n.s.^	-116° **
'fixed direction'	502 + 590	binocular	12	0.49	94°	0.64**	
	502 + 590	monocularly right-eyed	12	0.84^n.s.^	78°	0.81***	-16° ^n.s.^
	502 + 590	monocularly left-eyed	12	0.65 ^n.s.^	66°	0.67**	-28° ^n.s.^

The column *Light *gives the wavelength of light; *N*, number of birds tested, *med. r*_b_, median of the vector lengths based on the three recordings per bird, with asterisks indicating significant differences by the Mann Whitney U-test to the binocular tests under the same light; *α_N_, r_N_*, direction and length of the grand mean vector, with asterisks at r_N _indicating significance by the Rayleigh Test [17]. The last two columns give the angular differences to the binocular tests under the same light conditions and indicate significant differences in variance by the Mann Whitney U-test. Significance levels: ***, p < 0.001; **, p < 0.01; *, p < 0.05; n.s., not significant.

**Table 2:** Compass orientation of the individual birds under turquoise light

	binocular			monocularly right-eyed			monocularly left eyed		
	
Bird	N	α_b_	r_b_	N	α_b_	r_b_	N	α_b_	r_b_
5	3	221°	0.36	3	5°	1.00	3	172°	0.28
6	3	29°	1.00	3	27°	0.31	3	348°	0.38
8	3	54°	0.56	3	9°	0.65	3	177°	0.39
17	3	7°	0.95	3	14°	0.28	3	322°	0.81
18	3	46°	0.30	3	10°	0.87	3	212°	0.38
19	3	358°	0.99	3	11°	0.73	3	184°	0.50
20	3	157°	0.84	3	350°	0.74	3	97°	0.18
22	3	20°	0.99	3	40°	1.00	3	356°	0.82
23	3	328°	0.67	3	345°	0.82	3	18°	0.34
24	3	29°	0.97	3	12°	0.58	3	244°	0.84
25	3	35°	0.98	3	2°	0.66	3	99°	0.76
26	3	354°	1.00	3	22°	0.96	3	322°	0.33

**Table 3:** 'Fixed direction' responses of the individual birds under turquoise-and-yellow light

	binocular			monocularly right-eyed			monocularly left eyed		
	
Bird	N	α_b_	r_b_	N	α_b_	r_b_	N	α_b_	r_b_
5	3	166°	0.07	3	164°	0.93	3	61°	0.94
6	3	141°	0.50	3	59°	0.35	2	17°	0.84
8	3	38°	0.60	3	79°	0.80	3	66°	0.76
17	3	60°	0.96	3	44°	0.78	3	68°	0.98
18	3	104°	0.28	3	97°	0.90	3	34°	0.54
19	3	88°	0.31	2	8°	0.74	3	97°	0.34
20	3	233°	0.99	3	123°	0.95	3	251°	0.33
22	3	123°	0.91	3	83°	0.87	3	120°	0.28
23	3	86°	0.47	3	65°	0.98	3	40°	0.40
24	3	82°	0.88	3	72°	0.30	3	126°	0.86
25	3	84°	0.40	3	70°	0.33	3	26°	0.38
26	3	21°	0.29	3	88°	0.98	3	87°	0.82

Monocularly right-eyed robins with their left eye covered showed no effect of the treatment under either light regime. Covering the right eye, in contrast, led to disorientation under turquoise light, while it had no effect on the robins' behavior under turquoise-and-yellow light.

In summary, while the compass orientation under turquoise light broke down when the birds had to rely on their left eye alone, the orientation under turquoise-and-yellow light remained unaffected.

## Discussion

Compass orientation again proved to be lateralized in favor of the right eye. So far, lateralization of the magnetic compass had only been demonstrated under 565 nm green light in robins [5,16] and under white light in Australian silvereyes, *Zosterops l. lateralis *[15], robins (unpublished), domestic chickens, *Gallus gallus *[18] and homing pigeons, *Columba livia domestica *[19] (but see [20]); the present data are the first obtained under monochromatic 502 nm turquoise light. As long as the magnetic compass is properly working, it appears to be lateralized in favor of the right eye, irrespective of the light regime. Where this lateralization occurs-at the periphery or in higher centers in the brain-is not yet known with certainty. The asymmetrical structures in the avian visual system (see e.g. [14,21,22]), however, make a lateralization in higher centers most likely.

'Fixed direction' responses, in contrast, do not seem to be lateralized-for them, the input of both eyes appears to be equal. This speaks against an interaction of the receptors in the beak with the radical pair mechanism in the right eye system for the manifestation of the 'fixed direction'; rather, the specific directions seem to arise from interactions of these magnetite-based receptors with visual input that is processed in a non-lateralized way. For example, at least in pigeons, feature-based object vision is lateralized towards the left hemisphere, while spatial orientation by using a global reference frame is not processed in an asymmetrical way. As a result, pigeons show right-eye superiority when using prominent landmarks for orientation, while they display no left-right differences when relying on a general spatial reference system [23,24].

The general influence of the visual system on magnetoreception is still poorly understood. Contour vision plays an important role in magnetoreception, probably in distinguishing modulations of activity caused by magnetic input of the radical pair processes from that of objects represented by visual input [16]. The light condition leading to a disruption of compass orientation and the emergence of 'fixed direction' responses do not seem to interfere with the radical pair processes underlying the compass themselves-the radical pair mechanism should still be functioning. We must assume that some processes in the visual system interact with the magnetic compass system at a higher level in the brain to deactivate the respective magnetic information (see [1] for discussion). In this case, however, the magnetoreception system in the eye is involved, with the respective directional information mediated by the optic nerve and processed by parts of the visual system (e.g. [25-27], so that interactions with visual input is easily conceivable.

Any information from the magnetite-based receptors in the beak, on the other hand, is transported to the brain by the ophthalmic nerve, part of the trigeminal system (e.g. [28-31]). The directing information from these receptors must be combined with visual input to produce the specific 'fixed direction' observed under the respective light regime. In birds, trigeminal information from the *nucleus principalis trigemini *of the brainstem directly reaches the *nucleus basorostralis *(Bas) of the telencephalon, bypassing the diencephalon [32-34]. After being processed within the Bas and its associative surrounding, trigeminal information is conveyed towards the *nidopallium caudolaterale *(NCL) and the *arcopallium intermedium *(AI) in the caudal telencephalon. Both NCL and AI also receive afferents from the telencephalic sources of the thalamo-and tectofugal visual systems [35]. One of the major descending motor pathways that controls important aspects of the movement patterns in birds arises from AI [33]. Thus, both NCL and AI could constitute the critical structures for the integration of magnetite-based trigeminal information and vision, reflected in the 'fixed direction' responses.

## Conclusion

Our data show that 'fixed direction' responses are not lateralized. This suggests that the interactions between the magnetite-receptors in the beak and the visual system occurring at higher levels in the brain, do not involve the magnetoreception system based on radical pair process in the right eye, but rather other, non-lateralized components of the visual system.

## Competing interests

The authors declare that they have no competing interests.

## Authors' contributions

RW conceived, designed and coordinated the study, participated in its performance and drafted the manuscript, DG and SD performed the experiments and contributed to the discussion, OG contributed substantially to the discussion, WW conceived and designed the study, participated in its performance and contributed substantially to the discussion. All authors read and approved the final manuscript.

## References

[B1] Wiltschko R Stapput K Thalau P Wiltschko W Directional orientation of birds by the magnetic field under different light conditions J R Soc Interface 2010 7 Suppl 2 S163 S177 10.1098/rsif.2009.0367.focus 19864263 PMC2843996

[B2] Ritz T Thalau P Philllips JB Wiltschko R Wiltschko W Resonance effects indicate a radical-pair mechanism for avian magnetic compass Nature 2004 7 177 180 10.1038/nature02534 15141211

[B3] Thalau P Ritz T Stapput K Wiltschko R Wiltschko W Magnetic compass orientation of migratory birds in the presence of a 1.315 MHz oscillating field Naturwissenschaften 2005 7 86 90 10.1007/s00114-004-0595-8 15614508

[B4] Ritz T Wiltschko R Hore PJ Rodgers CT Stapput K Thalau P Timmel CR Wiltschko W Magnetic compass of birds is based on a molecule with optimal directional sensitivity Biophys J 2009 7 3451 3457 10.1016/j.bpj.2008.11.072 19383488 PMC2718301

[B5] Wiltschko W Traudt J Güntürkün O Prior H Wiltschko R Lateralisation of magnetic compass orientation in a migratory birds Nature 2002 7 467 470 10.1038/nature00958 12368853

[B6] Wiltschko R Ritz T Stapput K Thalau P Wiltschko W Two different types of light-dependent responses to magnetic fields in birds Curr Biol 2005 7 1518 1523 10.1016/j.cub.2005.07.037 16111946

[B7] Fleissner G Holtzkamp-Rötzler E Hanzlik M Winklhofer M Fleissner G Petersen N Wiltschko W Ultrastructural analysis of a putative magnetoreceptor in the beak of homing pigeons J Comp Neuro 2003 7 350 360 10.1002/cne.10579 12619070

[B8] Falkenberg G Fleissner G Schuchardt K Kuehbacher M Thalau P Mouritsen H Heyers D Wellenreuther G Fleissner G Avian magnetoreception: elaborate iron mineral containing dentrides in the upper beak seem to be a common feature of birds PLoS One 2010 7 e9231 10.1371/journal.pone.0009231 20169083 PMC2821931

[B9] Wiltschko R Stapput K Ritz T Thalau P Wiltschko W Magnetoreception in birds: different physical processes for two types of directional responses HFSP Journal 2007 7 41 47 10.2976/1.2714294 19404459 PMC2645559

[B10] Stapput K Thalau P Wiltschko R Wiltschko W Orientation of birds in total darkness Curr Biol 2008 7 602 606 10.1016/j.cub.2008.03.046 18424144

[B11] Wiltschko W Munro U Ford H Wiltschko R Bird navigation: what type of information does the magnetite-based receptor provide? Proc R Soc Lond B 2006 7 2815 2820 10.1098/rspb.2006.3651 PMC1664630 17015316

[B12] Wiltschko W Wiltschko R Magnetoreception in birds: two receptors for two different tasks J Ornithol 2007 7 Suppl 1 S61 S76 10.1007/s10336-007-0233-2

[B13] Wiltschko W Gesson M Stapput K Wiltschko R Light-dependent magnetoreception in birds: interaction of at least two different receptors Naturwissenschaften 2004 7 130 13 10.1007/s00114-003-0500-x 15034663

[B14] Vallortigara G Rogers L Survival with an asymmetrical brain: Advantages and disadvantages of cerebral lateralization Behav Brain Sci 2005 7 575 633 16209828 10.1017/S0140525X05000105

[B15] Wiltschko W Munro U Ford H Wiltschko R Lateralisation of magnetic compass orientation in Silvereyes, *Zosterops lateralis* Austr J Zool 2003 7 597 602 10.1071/ZO03022

[B16] Stapput K Güntürkün O Hoffmann KP Wiltschko R Wiltschko W Magnetoreception of directional information in birds requires non-degraded vision Curr Biol 2010 7 1259 1262 10.1016/j.cub.2010.05.070 20619654

[B17] Batschelet E Circular Statistics in Biology 1981 New York: Academic Press

[B18] Rogers LJ Munro U Freire R Wiltschko R Wiltschko W Lateralized response of chicks to magnetic cues Behav Brain Res 2008 7 66 71 10.1016/j.bbr.2007.07.029 17765981

[B19] Wilzeck C Wiltschko W Güntürkün O Wiltschko R Prior H Lateralization of magnetic compass orientation in pigeons J R Soc Interface 2010 7 Suppl 2 S131 S289 20053653 10.1098/rsif.2009.0436.focus PMC2843993

[B20] Hein CM Zapka M Heyers D Kutzschbauch S Schneider NL Mouritsen H Night-migratory garden warblers can orient with their magnetic compass using the left, the right or both eyes J R Soc Interface 2010 7 Suppl 2 S227 S233 10.1098/rsif.2009.0376.focus 19889693 PMC2844002

[B21] Rogers LJ Behavioral, structural and neurochemical asymmetries in the avian brain: a model system for studying visual development and processing Neurosci Biobehav Rev 1996 7 487 503 10.1016/0149-7634(95)00024-0 8880736

[B22] Güntürkün O Avian visual lateralization: a review NeuroReport 1997 7 3 11 9172127

[B23] Prior H Güntürkün O Parallel working memory for spatial location and object-cues in foraging pigeons. Binocular and lateralized monocular performance Learn Memory 2001 7 44 51 10.1101/lm.36201 PMC311357 11160763

[B24] Prior H Lingenauber F Nitschke J Güntürkün O Orientation and lateralized cue use in pigeons navigating a large indoor environment J Exp Biol 2002 7 1795 1805 12042338 10.1242/jeb.205.12.1795

[B25] Mai JK Semm P Pattern of brain glucose utilization following magnetic stimulation J Hirnforsch 1990 7 331 336 2230101

[B26] Heyers D Manns M Luksch H Güntürkün O Mouritsen H A visual pathway links brain structures active during magnetic compass orientation in migratory birds PlosOne 2007 7 e937 10.1371/journal.pone.0000937 PMC1976598 17895978

[B27] Zapka M Heyers D Hein SM Engels S Schneider NL Hans J Weiler S Dreyer D Kishkinev D Wild JM Mouristen H Visual but not trigeminal mediation of magnetic compass information in a migratory bird Nature 2009 7 1274 1277 10.1038/nature08528 19865170

[B28] Semm P Beason RC Responses to small magnetic variations by the trigeminal system of the Bobolink Brain Res Bull 1990 7 735 740 10.1016/0361-9230(90)90051-Z 2289162

[B29] Beason RC Semm P Does the avian ophthalmic nerve carry magnetic navigational information? J Exp Biol 1996 7 1241 1244 9319100 10.1242/jeb.199.5.1241

[B30] Mora CV Davison M Wild JM Walker M Magnetoreception and its trigeminal mediation in the homing pigeon Nature 2004 7 508 511 10.1038/nature03077 15565156

[B31] Heyers D Zapka M Hoffmeister M Wild JM Mouritsen H Magnetic field changes activate the trigeminal brainstem complex in a migratory bird Proc Natl Acad Sci USA 2010 7 9394 9399 10.1073/pnas.0907068107 20439705 PMC2889125

[B32] Wallenberg A Der Ursprung des Tractus isthmo-striatus (oder bulbo-striatus) der Taube Neurol Zentralbl 1903 7 98 101

[B33] Wild JM Arends JJA Zeigler HP Telencephalic connections of the trigeminal system in the pigeon (*Columba livia*): A trigeminal sensormotor circuit J Comp Neurol 1985 7 441 464 10.1002/cne.902340404 3988994

[B34] Schall U Güntürkün O Delius JD Sensory projections to the nucleus basalis prosencephali of the pigeon Cell Tissue Res 1986 7 539 546 10.1007/BF00218555 2428501

[B35] Kröner S Güntürkün O Afferent and efferent connections of the caudolateral neostriatum in the pigeon (*Columba livia*): A retro-and anterograde pathway tracing study J Comp Neurol 1999 7 228 260 10.1002/(SICI)1096-9861(19990503)407:2<228::AID-CNE6>3.0.CO;2-2 10213093

